# Microstructural Evolution and Mechanical Properties of AE45-Gd Magnesium Alloy Under Different Aging Processes

**DOI:** 10.3390/ma19143098

**Published:** 2026-07-19

**Authors:** Dengyun Lei, Jiyuan Li, Yulong He, Monan Xue, Siqi Yang, Huisheng Cai

**Affiliations:** Inner Mongolia Key Laboratory of New Materials and Surface Engineering, School of Material Science and Engineering, Inner Mongolia University of Technology, Hohhot 010051, China; 202320410012@imut.edu.cn (D.L.); 20241800354@imut.edu.cn (J.L.); 20251100237@imut.edu.cn (Y.H.); 20251800355@imut.edu.cn (M.X.); 20241800329@imut.edu.cn (S.Y.)

**Keywords:** AE45-Gd magnesium alloy, conventional aging, aging under pressure, microstructure, mechanical properties

## Abstract

**Highlights:**

- The effects of conventional aging and aging under pressure on the strain-hardening behavior of AE45-Gd magnesium alloy were investigated.
- The Ostwald ripening phenomenon during the aging process was discovered, and aging under pressure will promote this phenomenon.
- Manganese segregates and enriches in Al_2_RE compounds, and such enrichment inhibits the growth of Al_2_RE compounds.

**Abstract:**

To investigate the microstructural evolution and mechanical property changes in an as-extruded AE45-Gd magnesium alloy subjected to different aging treatments, an alloy containing multi-scale second phases was prepared by extrusion and subsequently subjected to artificial aging and aging under pressure. The microstructural evolution, age-hardening behavior, and mechanical properties of the alloy under different aging treatments were investigated. It was found that conventional isothermal aging can improve the mechanical properties to a certain extent. As the aging temperature increases, the peak-aged hardness decreases slightly, while the time to reach peak aging is progressively shortened. Aging under pressure also significantly shortens the time to peak aging, though the peak-aged hardness changes little. The multi-scale second phases in the alloy, consisting of coarse blocky Al_2_RE (La, Ce, Gd) and fine granular Al_11_RE_3_ (La, Ce, Gd), exhibit typical Ostwald ripening during aging, and aging under pressure accelerates this process. The optimum aging parameters are determined to be 200 °C for 16 h. After this treatment, the alloy achieves a yield strength of 183.95 MPa, a tensile strength of 322.50MPa, and an elongation of 4.32%. Microcracks predominantly initiate at the large Al_2_RE (La, Ce, Gd) compounds. The small granular Al_11_RE_3_ (La, Ce, Gd) particles show little evidence of self-cracking, and cracking mainly occurs at the phase boundaries between these particles and the magnesium matrix.

## 1. Introduction

Driven by the global energy transition and the “dual carbon” strategic goals, light-weighting has become one of the core technological pathways for achieving energy savings and emission reduction in the transportation sectors (automotive and aerospace). Magnesium alloys, as one of the lightest metallic structural materials, are hailed as the “green engineering materials of the 21st century” owing to their low density, high specific strength, and excellent castability, thereby exhibiting great application potential [1,2,3]. Among them, Mg-Al-based high-pressure die casting (HPDC) magnesium alloys have emerged as highly promising structural materials because of their favorable room-temperature strength, good fluidity during casting, high damping capacity, and good machinability [4,5].

Conventional Mg-Al-based alloys (e.g., AZ91, AM60) suffer a drastic decline in mechanical properties, particularly creep resistance, when exposed to service temperatures exceeding 120 °C, which severely restricts their application in high-temperature conditions [6]. The root cause of this performance bottleneck lies in microstructural instability: the β-Mg_17_Al_12_ phase, which is distributed as a continuous network along grain boundaries, softens at elevated temperatures and fails to effectively pin the grain boundaries, thereby making grain boundary sliding the dominant creep failure mechanism [7]. To overcome this challenge, researchers have added rare earth (RE) elements into the Mg-Al alloy, developing heat-resistant magnesium alloys represented by AE42 [8] and AE44 [9]. The addition of RE elements effectively suppresses the precipitation of the Mg_17_Al_12_ phase, leading instead to the formation of Al-RE intermetallic compounds (e.g., Al_11_RE_3_, Al_2_RE) with higher thermal stability. These phases are dispersedly distributed along grain boundaries, significantly enhancing the high-temperature stability and creep resistance of the grain boundaries [10].

In the as-cast condition, the microstructure of AE44 magnesium alloy is primarily composed of α-Mg matrix and intermetallic compounds distributed along the grain boundaries. However, the most prominent issue is the formation of coarse, acicular or lath-shaped Al_11_RE_3_ phases [11]. Under loading, the tips of these Al_11_RE_3_ phases act as stress concentration sites, readily initiating microcrack nucleation. During tensile or creep deformation, cracks preferentially propagate along these brittle phases, leading to premature brittle fracture of the alloy and consequently a significant reduction in both strength and ductility [12].

To address the above issues, the addition of the rare earth element Gd has been demonstrated to be an effective optimization strategy. The addition of Gd reacts with Al in the alloy to form a new, more stable second phase, Al_2_Gd [13], thereby suppressing the formation of coarse acicular Al_11_RE_3_ phases. Gd possesses a high solid solubility in magnesium alloys [14], which means that a substantial amount of Gd atoms can dissolve into the α-Mg matrix. During the subsequent aging process, numerous Gd-containing nanoscale precipitates are formed from the matrix. These dispersedly distributed precipitates can effectively pin dislocations, generating strong precipitation strengthening and thereby significantly enhancing the strength of the alloy [10].

Beyond compositional optimization, hot extrusion [15] is an essential plastic deformation process for further enhancing the comprehensive properties of AE44 magnesium alloy. Su et al. [16] investigated the effects of Gd and Y additions on the age-hardening behavior and mechanical properties of an extruded Mg-Gd-Zn-Mn alloy. The results indicated that the combined process of extrusion and aging (E-A, extruded-T5) can significantly enhance the strength of the alloy. This is because the intense shear stress during extrusion effectively breaks down the coarse Al_11_RE_3_ phase in the as-cast microstructure, making it finer and more dispersedly distributed in a streamlined pattern along the extrusion direction, thereby substantially alleviating its deleterious effect on matrix integrity. Meanwhile, the mechanical properties of the alloy are significantly improved after aging treatment. The choice of peak-aging temperature directly determines the size and number density of the precipitates and thus affects the yield strength, tensile strength, and even fracture toughness of the alloy [17,18]. Improper aging processes may lead to precipitate coarsening or discontinuous precipitation along grain boundaries, thereby impairing the ductility of the material. In addition, the evolution of the multi-scale second phases in the alloy during aging is not well understood.

Artificial aging is a core heat treatment technique for tailoring the precipitation phases and optimizing the mechanical properties of rare earth magnesium alloys. Conventional stress-free aging often suffers from slow precipitation kinetics and an excessively long period to reach peak aging [19]. Applying a constant compressive stress during aging can introduce additional strain energy via lattice distortion, thereby reducing the diffusion activation energy of solute atoms and the nucleation barrier of precipitates, and substantially shortening the holding time required to attain peak aging [20]. However, the age-hardening behavior of magnesium alloys during aging under pressure remains unclear.

Based on this, the present study focuses on an as-extruded AE45-Gd magnesium alloy, establishing a microstructure featuring multi-scale second phases, and subjecting the alloy to different aging treatments. The effects of aging parameters (temperature, time, and pressure) on the microstructural evolution and age-hardening behavior are investigated. The mechanical properties of the alloy and the role of the multi-scale second phases in the fracture process under loading are analyzed.

## 2. Materials and Methods

In this study, AE45 magnesium alloy and Mg-30%Gd master alloy were selected as raw materials. The AE45 magnesium alloy was melted in a crucible resistance furnace. During melting, argon gas was continuously introduced into the crucible to prevent burning and oxidation of the magnesium alloy. After the alloy was completely melted, the temperature was further raised to 720 °C, and the Mg-30%Gd master alloy was added according to the designed composition of the experimental alloy. Once the master alloy was melted, the melt was thoroughly stirred, then subjected to degassing and refining to remove slag from the melt. Subsequently, the melt was held for 10 min. When the melt temperature dropped to 700 °C, the melt was poured into a metal mold preheated to 250 °C to complete the casting. The as-cast alloy has a diameter of 60 mm and a height of 150 mm. The chemical composition of the alloy was determined by inductively coupled plasma optical emission spectrometry (ICP-OES), and the results are presented in Table 1.

The as-cast AE45-Gd magnesium alloy was machined into a cylinder with a diameter of 60 mm and a height of 80 mm, homogenized at 350 °C for 3 h [21], and subsequently extruded into a solid bar with a diameter of 12 mm and a length of 2000 mm using a 200-ton four-column hydraulic press at an extrusion speed of 2 m/min and with the extrusion die preheated to 300 °C.

To further improve the mechanical properties of the as-extruded alloy, aging treatments with different process parameters were conducted. The conventional aging process involved holding at 180 °C, 200 ℃, and 220 ℃ for up to 24 h, respectively. The hardness of the alloy was measured at various aging times to construct age-hardening curves, and the age-hardening behavior of the alloy during conventional aging was analyzed. Meanwhile, a pressurizing device was independently designed for this experiment: it consisted of two M20 × 30 304 heat-resistant stainless steel bolts and an M20 × 2.5 internally threaded base. The specimen was placed on the flat surface of the lower bolt, and torque was applied by rotating the upper bolt. The torque was converted into a vertical compressive force through the threads, thereby applying different pressure levels to the alloy. Subsequently, the alloy subjected to a certain pressure underwent artificial aging treatment. A digital torque wrench with an accuracy of 0.01 N·m was used to apply torque to the bolts. Different torque levels were applied to impose different pressures on the alloy.

**Figure 1:**
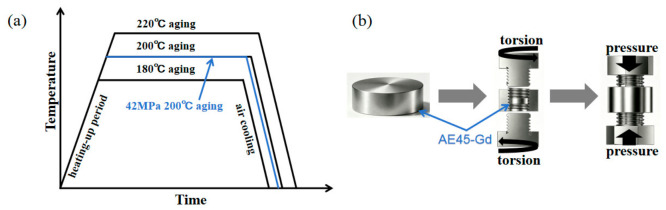
(**a**) Aging process schematic, (**b**) pressurizing device schematic.

According to the VDI 2230 standard [22] of the Association of German Engineers, the relationship between the axial compressive force FM and the tightening torque MA can be calculated by the following equation:

(1) $$\begin{array}{c} \begin{array}{c} M_{A}\;=\;F_{M}\left(0.16\;P\;+\;0.58\;d_{2}\;\mu_{G}\;+\;\mu_{K}\cdot \frac{D_{\mathrm{Km}}}{2}\right) \end{array} \end{array}$$

where *P* is the thread pitch (2.5 mm), *d*_2_ is the pitch diameter of the thread (18.3 mm), *μ_G_* and *μ_K_* are the coefficients of friction of the thread pair and the bearing surface, respectively (*μ* = 0.2), and *D_Km_* is the effective friction diameter of the bearing surface (30 mm). The aging processes applied to the experimental alloy are shown in Figure 1. The dimensions of the aged alloy specimens are 12 mm in diameter and 5 mm in height.

The phase constitution of the experimental alloy was analyzed using a Rigaku/SmartLab multifunctional X-ray diffractometer (Manufacturer: Rigaku Corporation, Akishima, Japan) at a scanning rate of 20°/min over a 2θ range of 20–80°. After grinding and polishing, the experimental alloy was etched with an Acetic-Picral etchant (50 mL ethanol + 2.5 g picric acid + 2.5 mL acetic acid + 50 mL deionized water). The microstructures of the as-cast, as-extruded, aged alloys, the room-temperature tensile fracture, and the cross-sectional microstructures perpendicular to the fracture were observed using an FEI-Apreo 2S HiVac field-emission scanning electron microscope (SEM) (Thermo Fisher Scientific, Hillsboro, OR, USA). Compositional analysis of the microstructures was carried out with an Oxford-Ultim Max 4.0 energy-dispersive spectrometer (EDS) (Oxford Instruments plc, High Wycombe, UK). 

**Figure 2:**
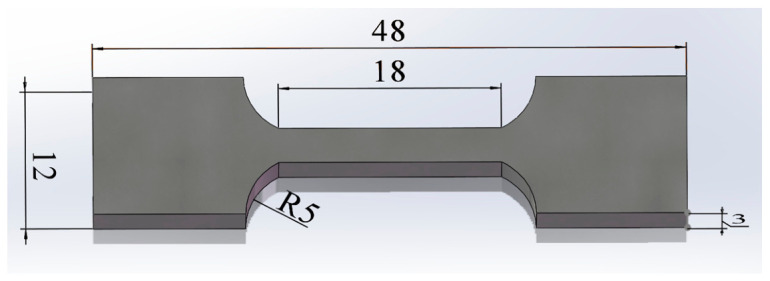
Dimensions of the room-temperature tensile specimen.

After grinding and polishing, the hardness of the alloy was measured using an HVS-1000A automatic turret digital microhardness tester (Shanghai Taiming Optical Instrument Co., Ltd., Shanghai, China) with a load of 49 N and a dwell time of 15 s. To ensure the accuracy of the experimental data, 20 hardness measurements were performed on each specimen, and the average value was taken as the hardness of the specimen. In accordance with the requirements of GB/T 228.1-2021 [23], the room-temperature tensile test of the alloy was conducted using an INSTRON/5982 dual-column floor-standing universal testing machine (Instron, Norwood, MA, USA) at a tensile speed of 2 mm/min. The tensile specimens were taken from the middle section of the extruded bars, and the orientation of the tensile specimens was parallel to the extrusion direction. At least three specimens were tested under each condition to ensure the accuracy of the experimental data, and the average values were taken as the tensile properties of the alloy. The dimensions of the tensile specimen are shown in Figure 2.

## 3. Results

### 3.1. Microstructure of AE45-Gd Magnesium Alloy

**Figure 3:**
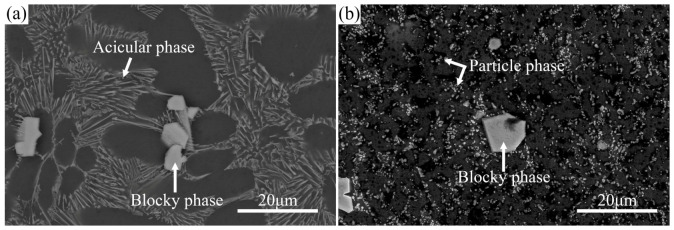
Microstructures of AE45-Gd alloys: (**a**) as-cast, (**b**) as-extruded.

Microstructures of the as-cast and as-extruded AE45-Gd alloy are shown in Figure 3. As shown in Figure 3a, the as-cast AE45-Gd alloy is mainly composed of the magnesium matrix, coarse blocky compounds, and acicular compounds. The microstructural characteristics of the alloy are similar to those reported by Wei J et al. [24], in which the compounds in the alloy mainly consist of acicular Al_11_RE_3_ and blocky Al_2_RE phases. After extrusion, the microstructure of the alloy underwent significant changes. The alloy is mainly composed of a magnesium matrix, large blocky compounds, and fine granular compounds. The large blocky compounds in the alloy did not change significantly; however, the acicular compounds disappeared and were completely transformed into fine granular compounds, and the grain size of the alloy also became finer. During extrusion, owing to the relatively large extrusion ratio (λ = 25), the microstructure of the as-extruded AE45-Gd alloy was considerably refined and more uniformly distributed [25,26,27].

**Figure 4:**
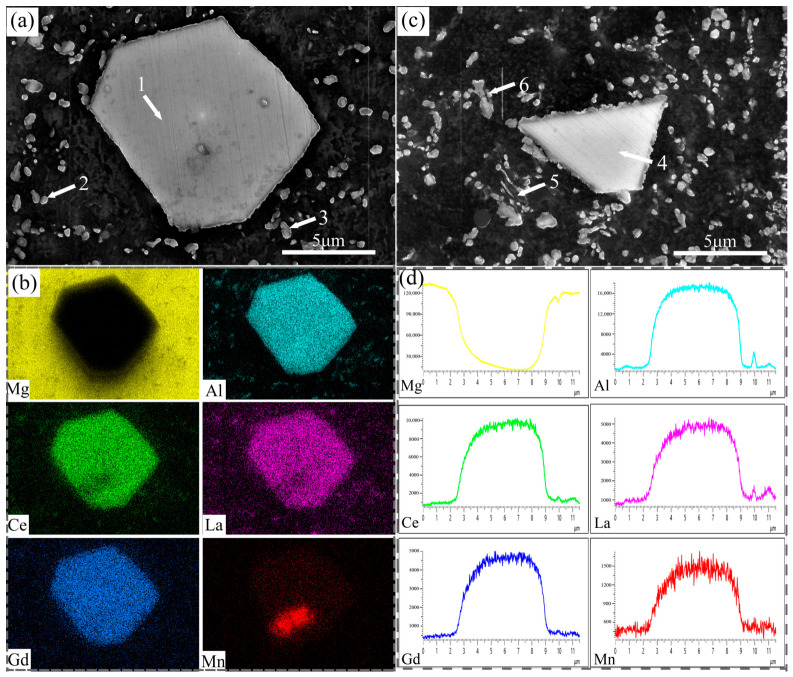
(**a**) SEM image of as-extruded AE45-Gd sample and EDS measurement positions, (**b**) EDS mapping, (**c**) SEM image of as-extruded AE45-Gd sample and EDS measurement positions, (**d**) EDS line scan results.

The EDS point analysis results reveal that the large blocky compounds are primarily composed of Al, La, Ce, and Gd, with an atomic ratio of Al:(La, Ce, Gd) close to 2:1, which is consistent with the compound reported in the literature [28]. Therefore, it can be inferred that the large blocky compound is Al_2_RE (La, Ce, Gd). Similarly, the small granular compounds are also mainly composed of Al, La, Ce, and Gd, and the Al:(La, Ce, Gd) atomic ratio is close to 11:3, suggesting that the small granular compound is Al_11_RE_3_ (La, Ce, Gd). The EDS point analysis results of the main compounds in the alloy are listed in Table 2. Due to the low solid solubility of RE elements in magnesium (<0.1 at.%) [29,30,31] and the larger electronegativity difference between Al and RE compared with that between Mg and RE, Al-RE compounds are more readily formed in Al-containing magnesium alloys. Meanwhile, the EDS mapping and line scan results show that the trace alloying element Mn in the alloy tends to segregate in the Al_2_RE (La, Ce, Gd) phase, as presented in Figure 4b,d. In Figure 4a, significant Mn segregation is observed below the blocky compound. At the location of pronounced Mn enrichment, clear signs of growth termination of the compound are evident, indicating that Mn segregation may inhibit compound growth, thereby altering the growth direction of the compound.

### 3.2. Age-Hardening Behavior of AE45-Gd Magnesium Alloy

**Figure 5:**
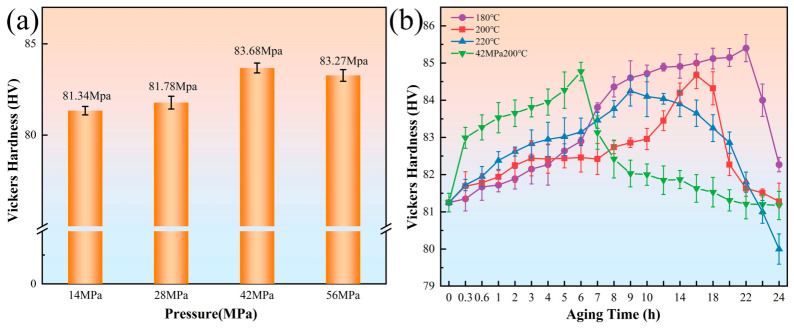
(**a**) Vickers hardness of AE45-Gd alloy after one hour of aging at 200 °C under different pressures. (**b**) Isothermal aging curves of AE45-Gd alloys.

Based on the aging processes for AE-series magnesium alloys and previous studies [32,33], the isothermal aging temperatures selected in this experiment were 180 °C, 200 °C, and 220 °C. To investigate the effect of the aging process on the age-hardening behavior of the alloy, aging under pressure was simultaneously carried out at 200 °C. In order to determine the applied pressure, aging treatments under different compressive stresses were first conducted at 200 °C. The hardness of the alloy after aging at 200 °C under different pressures for 1 h is shown in Figure 5a. It can be seen from the figure that following pressurized aging at 200 °C for 1 h, the alloy achieved the maximum hardness under an applied pressure of 42 MPa, without noticeable plastic deformation. Although the hardness degradation was not significant at 56 MPa, a further increase in pressure could induce plastic deformation of the specimens during aging, which would compromise the experimental results. Therefore, 42 MPa was ultimately selected as the optimal applied pressure. Accordingly, the pressure for the pressurized aging process was finally determined to be 42 MPa. The age-hardening curves of the alloy after different aging treatments are shown in Figure 5b. It can be observed that the hardness of the alloy increases gradually with prolonged aging time. The higher the aging temperature, the shorter the time to reach peak aging, although the peak-aged hardness decreases slightly. The peak hardness for aging at 200 °C is 84.67 HV, while the peak hardness for aging under 42 MPa at 200 °C is 84.77 HV. Compared with conventional aging, although the peak-aged hardness under pressure changes little, the time to reach peak aging is substantially shortened. For conventional aging at 200 °C, the time to peak aging is 16 h, whereas it is reduced to 6 h under 42 MPa at 200 °C, representing a 62.5% reduction. Furthermore, during conventional artificial aging, the hardness of AE45-Gd magnesium alloy increases slowly with aging time at the initial stage. However, during aging under pressure, the hardness can reach a relatively high value within 20 min, with a significantly shortened incubation period and a more rapid age-hardening response. The peak-aged hardness and the time to reach peak aging of AE45-Gd magnesium alloy under different aging processes are listed in Table 3.

As shown in Figure 5b, the hardness of the alloy decreases rapidly after reaching peak aging under the various aging processes. In light of the Friedel cutting mechanism and the Orowan bypassing mechanism [34,35], this behavior is likely due to the ongoing nucleation and coarsening of precipitates within the alloy. When the alloy reaches peak aging, the precipitates are relatively fine and dispersedly distributed in the matrix, and they can be cut through by dislocations; after peak aging, the precipitates coarsen and become impenetrable obstacles, exerting a certain pinning effect and causing the dislocation–obstacle interaction to transition from the Friedel cutting mechanism to the Orowan bypassing mechanism. Since the energy barrier required for the Orowan bypassing mechanism is lower than that for the Friedel cutting mechanism, dislocations move predominantly by the cutting mechanism before peak aging; after peak aging, as the precipitates coarsen, dislocations move predominantly by the bypassing mechanism, resulting in a decrease in yield stress, which is macroscopically manifested as a decline in hardness.

### 3.3. Microstructure of AE45-Gd Magnesium Alloy Under Different Aging Processes

**Figure 6:**
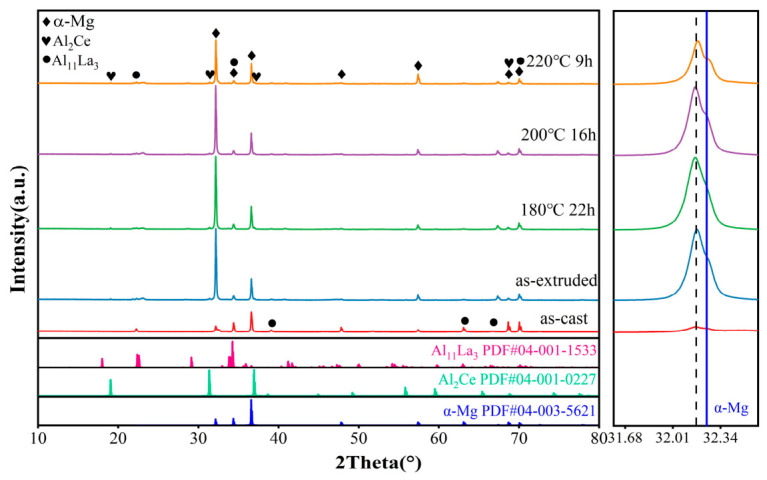
XRD patterns of as-cast, as-extruded AE45-Gd alloy and the alloy after aging.

The XRD spectra and phase identification results of the AE45-Gd magnesium alloy in the as-cast, as-extruded, and peak-aged conditions are shown in Figure 6. Comparison with standard PDF cards reveals that the AE45-Gd magnesium alloy contains only diffraction peaks of the α-Mg phase, Al_2_Ce phase, and Al_11_La_3_ phase. As indicated by the EDS analysis results above, La, Ce, and Gd elements all enter into the Al_2_Ce and Al_11_La_3_ phases, forming Al_2_RE (La, Ce, Gd) and Al_11_RE_3_ (La, Ce, Gd) phases. Meanwhile, it is readily apparent that distinct diffraction peaks of the Al_11_RE_3_ (La, Ce, Gd) phase are present in the as-cast AE45-Gd magnesium alloy, whereas their intensity is very low in the as-extruded and peak-aged alloys, which corresponds to the microstructural characteristics of the alloy. After extrusion, the Al_11_RE_3_ (La, Ce, Gd) phase in the AE45-Gd magnesium alloy transforms from relatively large acicular phases into fine granular phases. This refinement leads to broadening and weakening of the diffraction peaks, and consequently no distinct diffraction peak of the Al_11_RE_3_ (La, Ce, Gd) phase appears in the XRD pattern of the as-extruded alloy. Furthermore, the 2θ angle of the α-Mg (100) plane in the AE45-Gd alloy is 32.17°, compared with 32.24° in the standard pattern, exhibiting a small shift of 0.07° towards lower angles. This indicates that a certain amount of La, Ce, and Gd rare earth elements are dissolved in the α-Mg matrix; however, owing to the low solid solubility of RE elements in magnesium [29,30,31], the angular offset is relatively limited.

**Figure 7:**
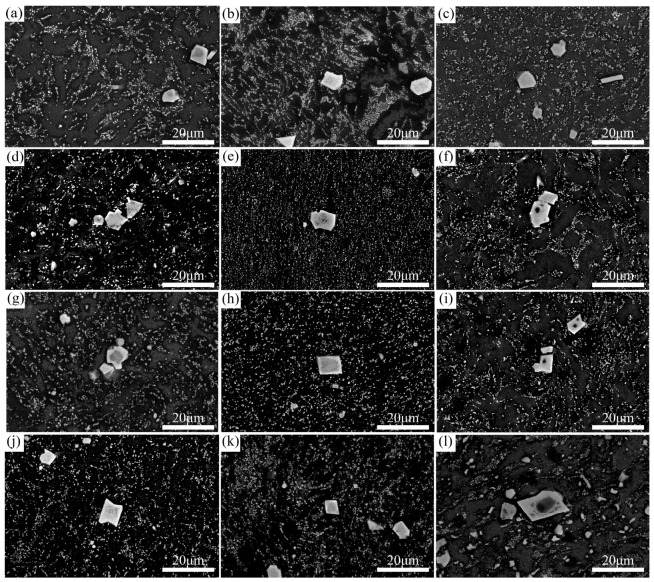
SEM image of AE45-Gd alloys after aging: (**a**) 180 °C 1 h, (**b**) 180 °C 22 h, (**c**) 180 °C 24 h, (**d**) 200 °C 1 h, (**e**) 200 °C 16 h, (**f**) 200 °C 24 h, (**g**) 220 °C 1 h, (**h**) 220 °C 9 h, (**i**) 220 °C 24 h. SEM image of AE45-Gd alloys after pressure aging: (**j**) 200 °C 1 h, (**k**) 200 °C 6 h, (**l**) 200 °C 24 h.

**Figure 8:**
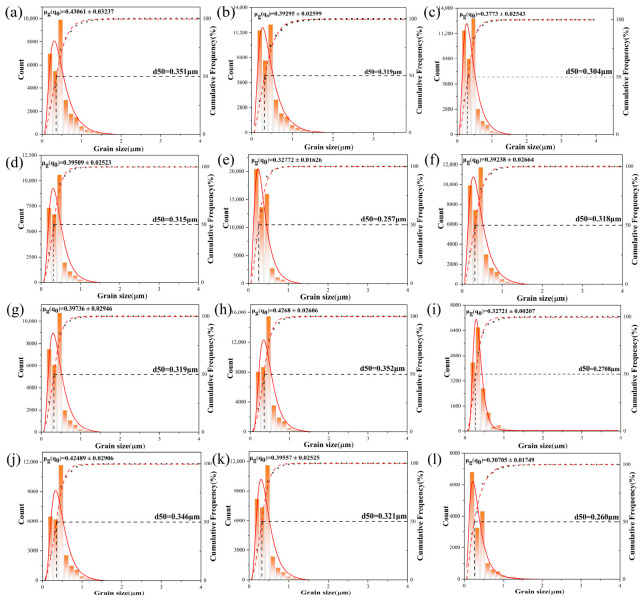
Size distribution of small-sized particulate phase in AE45-Gd alloys after aging: (**a**) 180 °C 1 h, (**b**) 180 °C 22 h, (**c**) 180 °C 4 h, (**d**) 200 °C 1 h, (**e**) 2000 °C 16 h, (**f**) 200 °C 24 h, (**g**) 220 °C 1 h, (**h**) 220 °C 9 h, (**i**) 220 °C 24 h. Size distribution ofAE45-Gd alloys after pressure aging: (**j**) 200 °C 1 h, (**k**) 200 °C 6 h, (**l**) 200 °C 24 h. Black scatter plots depict the cumulative frequency of particle sizes in ascending order on the X-axis; the red dashed curve corresponds to the Boltzmann function fit.

**Figure 9:**
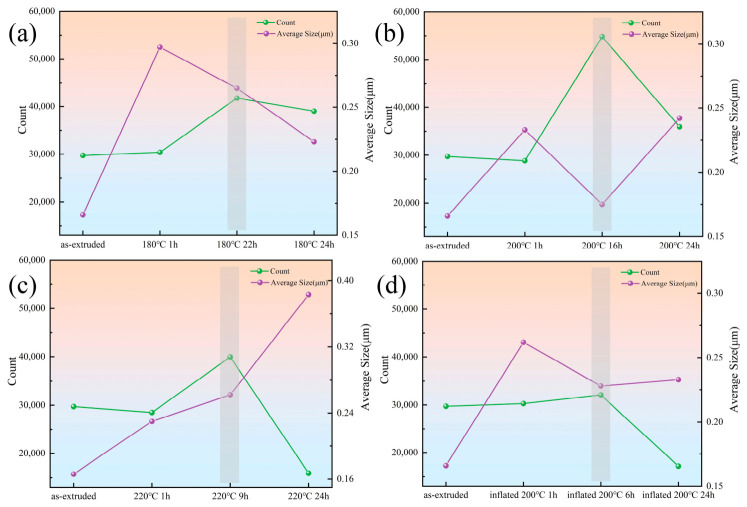
The number and the average size of small-sized particulate phase in extruded and aged conditions. (**a**) aged at 180°C, (**b**) aged at 200 °C, (**c**) aged at 220°C, (**d**) aged at 200°C under 42MPa. The shaded area indicates the peak-aging position.

The microstructures of the AE45-Gd magnesium alloy in the under-aged, peak-aged, and over-aged conditions after different aging heat treatments are shown in Figure 7. Similar to the microstructure of the as-extruded alloy, the alloy is primarily composed of a magnesium matrix, large blocky Al_2_RE (La, Ce, Gd) compounds, and small granular Al_11_RE_3_ (La, Ce, Gd) particles. The large Al_2_RE (La, Ce, Gd) compounds did not change significantly with prolonged aging time or increasing aging temperature. However, the size and number of the small Al_11_RE_3_ (La, Ce, Gd) particles changed markedly as aging progressed. Using the WEKA model in ImageJ software (1.54i) combined with a Python (3.12.7) program for machine learning, the phases of different sizes in the SEM images were classified, and the number and size of the Al_11_RE_3_ (La, Ce, Gd) particles in the alloy were statistically analyzed. The variation trends are shown in Figure 8 and Figure 9. Overall, the number of Al_11_RE_3_ (La, Ce, Gd) particles reached its maximum, and their average size was relatively the smallest at the peak-aged condition. After conventional aging at 200 °C for 16 h, the Al_11_RE_3_ (La, Ce, Gd) particles were most uniformly and dispersedly distributed, with an average size of approximately 0.15 μm, providing a pronounced dispersion-strengthening effect on the alloy. After over-aging, the Al_11_RE_3_ (La, Ce, Gd) particles coarsened, and their number tended to decrease.

Furthermore, aging under pressure directly influences the number, size, and distribution of the Al_11_RE_3_ (La, Ce, Gd) particles in the alloy. After aging at 200 °C under 42 MPa, the number of Al_11_RE_3_ (La, Ce, Gd) particles is markedly lower than that after conventional aging at 200 °C, and their size is correspondingly larger. In the over-aged condition, the number of small Al_11_RE_3_ (La, Ce, Gd) particles decreases significantly, accompanied by the appearance of coarse blocky compounds.

### 3.4. Ostwald Ripening Phenomenon of AE45-Gd Magnesium Alloy During Aging

**Figure 10:**
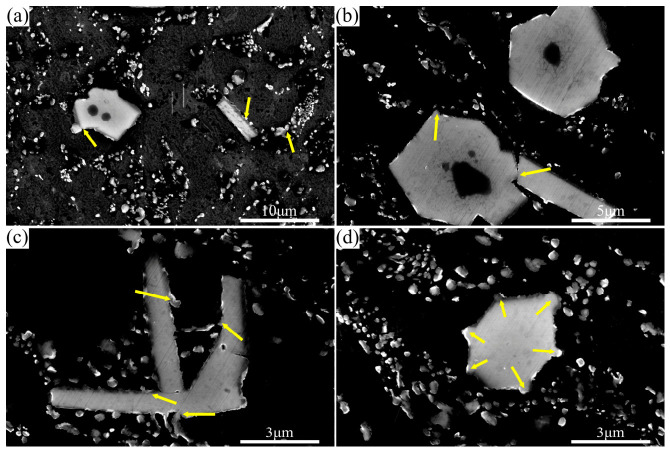
Typical Ostwald ripening phenomena during aging. (**a**) 200 °C 24 h, (**b**) 220 °C 24 h, (**c**) 220 °C 24 h, (**d**) 220 °C 24 h. Yellow arrows point to the engulfment of small particles by large particles.

In all the aged alloys, it was observed that the number of small Al_11_RE_3_ (La, Ce, Gd) particles decreased as aging progressed. During the microstructural examination of the aged alloys, phenomena such as larger second-phase particles engulfing smaller ones, as well as the bridging and coalescence of small particles, i.e., Ostwald ripening, were observed. In the over-aged alloys, it was clearly evident that the small second-phase particles dispersedly distributed around the large second-phase particles were significantly reduced. Typical Ostwald ripening phenomena during aging are shown in Figure 10. From the SEM images in Figure 7 and Figure 10, it can be seen that Ostwald ripening (OR) [36] occurred during the aging treatment, and this phenomenon was more pronounced during aging at 200 °C and 220 °C. In particular, irregularly shaped blocky Al_2_RE (La, Ce, Gd) phases can be clearly observed in the over-aged microstructure.

During aging, the small particles, possessing a larger specific surface area and higher interfacial energy, exhibit poor thermodynamic stability; in contrast, the larger particles have a smaller specific surface area and lower interfacial energy, and the system spontaneously evolves towards a state of minimum total interfacial energy. In the early stage of aging of the as-extruded alloy, a large number of submicron-sized fine Al_11_RE_3_ (La, Ce, Gd) particles precipitate uniformly in the matrix. At this stage, the particle size is small, the size distribution is uniform, and the concentration gradient between particles is weak, so Ostwald ripening scarcely occurs. When the alloy reaches peak aging, the nucleation of precipitates is essentially complete, the degree of particle dispersion is the highest, and the strengthening effect is optimal. When the aging temperature is too high or the holding time exceeds the peak-aging duration, the system enters the over-aging stage, where Ostwald ripening becomes the dominant mechanism of microstructural evolution, and the hardness of the alloy declines rapidly. Ostwald ripening is a phenomenon driven by the reduction of total interfacial energy, in which small particles dissolve and large particles grow. It causes coarsening of the strengthening phase and an increase in interparticle spacing, thereby weakening the dispersion strengthening effect and reducing the hardness and strength of the alloy. This is the core microstructural mechanism responsible for the continuous deterioration of mechanical properties after the alloy enters the over-aging stage.

### 3.5. Room Temperature Mechanical Properties of AE45-Gd Magnesium Alloy

**Figure 11:**
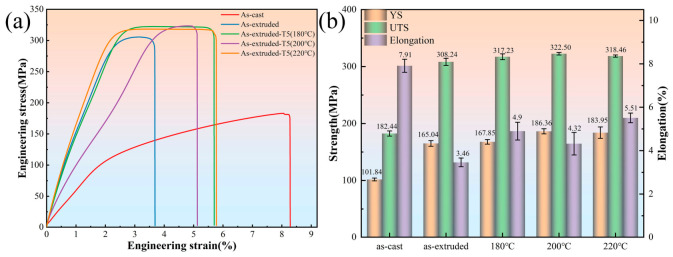
As-extruded and as-aged AE45-Gd alloys. (**a**) Engineering stress–engineering strain curves. (**b**) Histogram of tensile performance (tensile yield strength, ultimate tensile strength and elongation).

Figure 11 presents the engineering stress–engineering strain curves, together with the yield strength, tensile strength, and elongation of the AE45-Gd magnesium alloy in the as-cast, as-extruded, and peak-aged conditions at different aging temperatures. As can be seen from Figure 11a, the as-cast AE45-Gd magnesium alloy exhibits relatively low tensile strength and yield strength but with good elongation, demonstrating superior plastic deformation capability compared with the as-extruded alloy and the alloy directly aged after extrusion.

As can be seen from the room-temperature tensile properties of the AE45-Gd alloy (Figure 11b), both the yield strength and tensile strength of the as-cast AE45-Gd alloy are substantially increased after extrusion. A comparison between the as-extruded AE45-Gd alloy and the peak-aged alloys reveals that the yield strength, tensile strength, and elongation are all improved to a certain extent compared with the as-extruded alloy. Among them, the AE45-Gd alloy exhibits the optimum mechanical properties after peak aging at 200 °C, achieving a yield strength of 186.36 (±4.5) MPa, which represents a 12.92% increase over the as-extruded alloy, and an ultimate tensile strength of 322.5 (±2.3) MPa, which is 4.63% higher than that of the as-extruded alloy. The yield strength is the critical stress at which macroscopic plastic deformation initiates in a material, and it is extremely sensitive to microscopic obstacles. The high-density Al_11_RE_3_ (La, Ce, Gd) particles formed after peak aging greatly increase the initial resistance to dislocation motion, thus leading to a substantial increase in yield strength.

### 3.6. Fracture Morphology of AE45-Gd Magnesium Alloy

**Figure 12:**
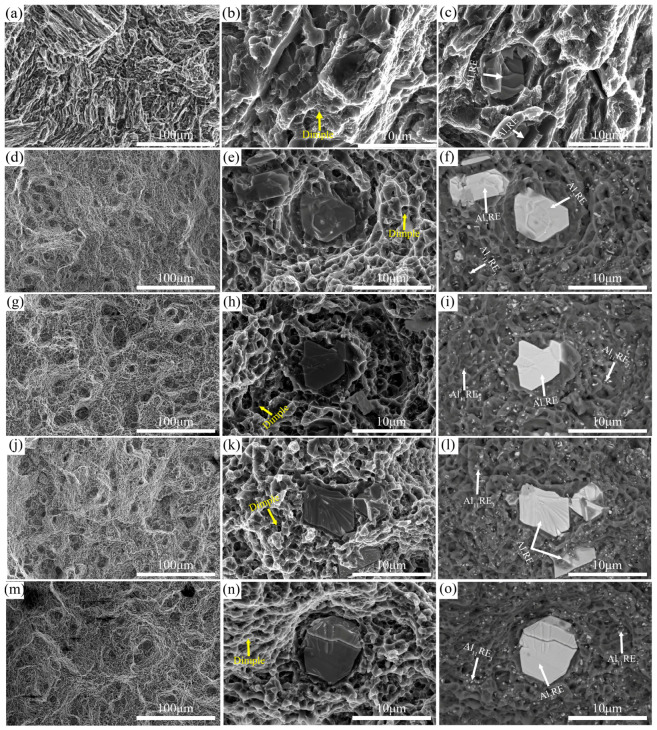
The fracture morphologies of AE45-Gd alloys: (**a**–**c**) as-cast; (**d**–**f**) as-extruded; (**g**–**i**) 180 °C peak-aged; (**j**–**l**) 200 °C peak-aged; (**m**–**o**) 220 °C peak-aged.

Figure 12 shows the fracture morphologies of the as-cast and as-extruded AE45-Gd magnesium alloy, as well as those after peak aging at 180 °C, 200 °C, and 220 °C. As can be seen, on the fracture surface of the as-cast alloy, fractured, blocky Al_2_RE (La, Ce, Gd) compounds and a large number of flattened dimples can be observed. After aging treatment, relatively large blocky Al_2_RE (La, Ce, Gd) compounds, small granular Al_11_RE_3_ (La, Ce, Gd) particles, and a small number of flattened dimples are present on the fracture. Meanwhile, the cleavage facets on the fracture are relatively small, and the fracture morphology is fine and uniform, which is closely related to the uniform and dispersive distribution of the small Al_11_RE_3_ (La, Ce, Gd) particles in the microstructure. The presence of numerous Al_2_RE (La, Ce, Gd) blocky compounds and Al_11_RE_3_ (La, Ce, Gd) granular particles on the fracture indicates that during the fracture process, cracks predominantly nucleate at the rare earth compounds, which also serve as critical nodes along the crack propagation path.

**Figure 13:**
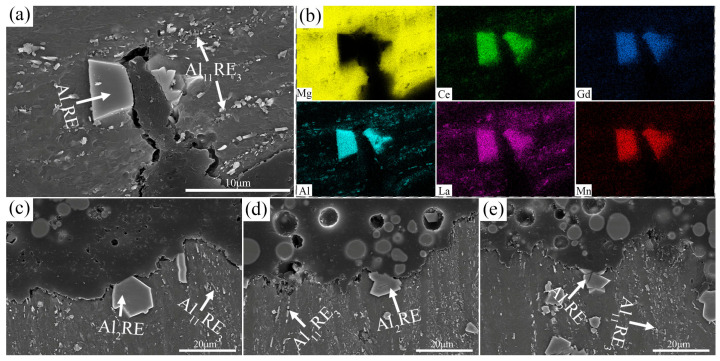
The cross-sectional microstructure perpendicular to the fracture and EDS mapping results of the fracture cross-section: (**a**) as-extruded, (**b**) EDS mapping results of as-extruded alloy, (**c**) 180 °C aged, (**d**) 200 °C aged and (**e**) 220 °C aged alloys.

The typical microstructure of the cross-section perpendicular to the fracture of the AE45-Gd magnesium alloy, together with the corresponding EDS mapping results, is shown in Figure 13. As can be seen, a large number of Al_2_RE (La, Ce, Gd) phases are distributed along the crack propagation path, and signs of cracking are also evident in the Al_2_RE (La, Ce, Gd) phases near the fracture. This observation indicates that microcracks primarily initiate at the Al_2_RE (La, Ce, Gd) phases during crack propagation, and these phases also serve as critical nodes along the propagation path. Meanwhile, Al_11_RE_3_ (La, Ce, Gd) particles can also be found along the main crack. Owing to their smaller size, these particles exhibit a much lower degree of cracking compared with the Al_2_RE (La, Ce, Gd) phases, and cracking predominantly occurs at the phase boundaries between the particles and the α-Mg matrix.

## 4. Discussion

### 4.1. Microstructural Evolution of the AE45-Gd Magnesium Alloy

**Figure 14:**
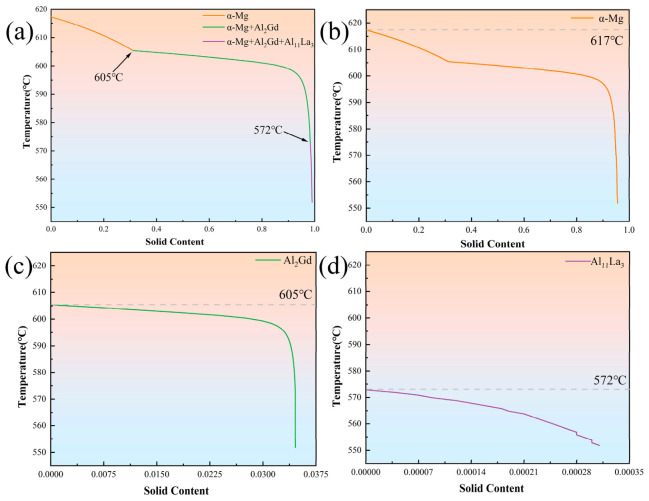
Solidification process of the AE45-Gd alloy calculated by Thermo-Calc: (**a**) AE45-Gd, (**b**) α-Mg, (**c**) Al_2_Gd, (**d**) Al_11_La_3_.

The non-equilibrium solidification process of the AE45-Gd magnesium alloy calculated using the Scheil–Gulliver (SG) model in Thermo-Calc software (2019a) is shown in Figure 14. As can be seen, during solidification, α-Mg first forms at 617 ℃, followed by the formation of the Al_2_RE compound when the temperature decreases to 605 ℃, and the Al_11_RE_3_ compound begins to form as the temperature further decreases to 572 ℃. According to the edge-to-edge matching (E2EM) model [37,38], the interatomic spacing misfit fr and the interplanar spacing misfit fd between the Al_11_RE_3_ and Al_2_RE phases can be calculated. Specifically, the fr for <112> Al_2_(RE)|[001] Al_11_RE_3_ is 3.89%; the fd values for {-222} Al_2_(RE)|(200) Al_11_RE_3_ and {-311} Al_2_(RE)|(200) Al_11_RE_3_ are 2.54% and 6.71%, respectively. These two compounds exhibit a certain crystallographic orientation relationship, and Al_2_RE forms prior to Al_11_RE_3_; therefore, Al_2_RE can serve as heterogeneous nucleation sites to refine and modify the Al_11_RE_3_ compound. Consequently, compared with AE-series magnesium alloys [39], the size of the Al_11_RE_3_ phase in the AE45-Gd magnesium alloy is relatively small.

After undergoing severe plastic deformation with a large extrusion ratio (25:1), dynamic recrystallization was triggered in the as-cast AE45-Gd magnesium alloy. The originally coarse as-cast grains were fragmented and recrystallized, resulting in a uniformly fine-grained microstructure. The acicular Al_11_RE_3_ (La, Ce, Gd) second phase in the as-cast alloy was crushed by the extrusion stress and transformed into fine granular particles. The as-extruded AE45-Gd magnesium alloy is thus composed of a magnesium matrix, small-sized Al_11_RE_3_ (La, Ce, Gd) second-phase particles, and large blocky Al_2_RE (La, Ce, Gd) phases. The as-extruded AE45-Gd magnesium alloy exhibits a typical multi-scale second-phase microstructure. After various aging treatments, the multi-scale second-phase microstructure of the alloy does not show significant changes. However, Ostwald ripening occurs in the alloy, leading to an increase in the size of the second phase.

**Figure 15:**
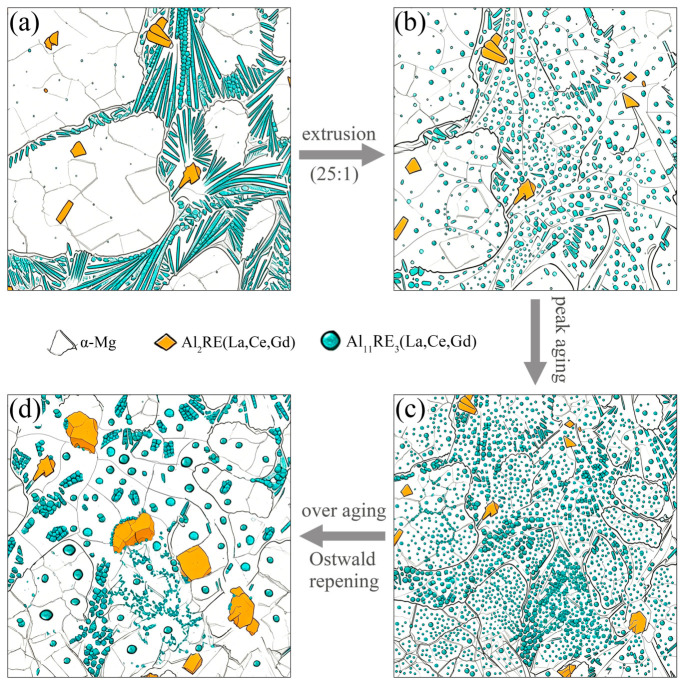
Microstructural transformation of AE45-Gd alloys: (**a**) as-cast, (**b**) as-extruded, (**c**) peak-aged state, (**d**) over-aging.

Direct aging treatment of the as-extruded AE45-Gd magnesium alloy revealed that, as aging progressed, the number of Al_11_RE_3_ (La, Ce, Gd) particles exhibited an overall trend of initially increasing and then decreasing, reaching a maximum at peak aging. After exceeding the peak aging stage, the Al_11_RE_3_ (La, Ce, Gd) particles gradually coarsened, and their number decreased, accompanied by a pronounced Ostwald ripening phenomenon. The small Al_11_RE_3_ (La, Ce, Gd) particles were progressively engulfed by the Al_2_RE (La, Ce, Gd) phase, or small particles bridged and coalesced to form larger particles. A schematic illustration of the microstructural evolution process of the AE45-Gd magnesium alloy from the as-cast to the as-extruded and then to the aged condition is shown in Figure 15.

### 4.2. Microstructural Evolution During Aging Under Pressure

During aging under pressure at 200 ℃ with an applied stress of 42 MPa, the time required for the AE45-Gd magnesium alloy to reach peak aging was significantly shortened. Under the pressurized condition, the alloy attained the peak-aged state within only 6 h, whereas conventional stress-free aging required up to 16 h to reach peak aging. This pronounced difference in aging kinetics is primarily attributed to the effect of the externally applied compressive stress.

The compressive stress continuously acting on the alloy maintains the system in a state of elevated strain energy [40], effectively reducing the diffusion activation energy of solute atoms and substantially accelerating the nucleation process of the Al_11_RE_3_ (La, Ce, Gd) compounds. Consequently, a large number of dispersedly distributed second-phase particles form within a short time, significantly shortening the incubation and precipitation period for peak aging and enhancing the age-hardening response of the alloy. However, the sustained stress combined with prolonged high-temperature holding simultaneously intensifies the Ostwald ripening behavior of the precipitates. In the pressurized aging system, the number fraction of small-sized Al_11_RE_3_ (La, Ce, Gd) particles decreases markedly. The fine particles continuously dissolve and are engulfed by the large second phases, or they bridge and coalesce with one another to form larger particles, ultimately resulting in a higher over-aging coarsening rate under pressurized aging than under conventional stress-free aging. With increasing conventional aging temperature or prolonged aging time, the size of the Al_11_RE_3_ (La, Ce, Gd) compounds gradually increases. Aging treatment of the alloy under a certain applied pressure accelerates the process of size enlargement of the Al_11_RE_3_ (La, Ce, Gd) compounds.

### 4.3. Fracture Mechanism of the As-Extruded AE45-Gd Magnesium Alloy

The fracture behavior of the AE45-Gd magnesium alloy is primarily governed by its microstructural condition. The Al_2_RE (La, Ce, Gd) phase, which is relatively large in size and exhibits poor deformability, readily develops strain mismatch with the adjacent α-Mg matrix when subjected to external loads. This strain incompatibility consequently results in localized stress concentration at the phase/matrix interfaces. When the local stress exceeds the interfacial bonding strength of the phase boundary or the fracture strength of the compound itself, cracks first initiate within the Al_2_RE (La, Ce, Gd) phase or at the phase boundary. Subsequently, adjacent microcracks interconnect and gradually develop into a main crack. In contrast, the Al_11_RE_3_ (La, Ce, Gd) particles are smaller in size and more dispersedly distributed, and their self-cracking tendency is significantly weaker than that of the coarse Al_2_RE (La, Ce, Gd) phase. During the fracture process, the Al_11_RE_3_ (La, Ce, Gd) particles more frequently exhibit cracking at the phase boundaries between themselves and the α-Mg matrix, rather than distinct fracture of the particles themselves.

The fracture mechanism of the AE45-Gd magnesium alloy is characterized as follows: microcracks initially form at the coarse blocky Al_2_RE (La, Ce, Gd) phases and their interfaces with the α-Mg matrix under external loading. With further deformation, these microcracks extend along the Al_2_RE phases and their phase boundaries and interconnect with the cracked regions at the phase boundaries of the Al_11_RE_3_ (La, Ce, Gd) particles, ultimately resulting in fracture failure of the alloy. The fracture behavior of the alloy is co-dominated by two mechanisms: cracking of the second phase itself and interfacial debonding between the second phase and the matrix. No significant variation in the fracture mechanism is observed in the as-extruded AE45-Gd magnesium alloy after various aging treatments. Crack initiation preferentially occurs at coarse second-phase particles, along with a small number of cracks at the interfaces between fine granular second-phase particles and the magnesium matrix.

## 5. Conclusions

This study systematically investigated the microstructural evolution and mechanical properties of the as-extruded AE45-Gd magnesium alloy under different aging processes. The main conclusions are as follows:

(1) Aging treatment of the as-extruded AE45-Gd magnesium alloy can improve its mechanical properties to a certain extent. As the aging temperature increases, the peak-aged hardness gradually decreases, but the time to reach peak aging is progressively shortened. Within the scope of this study, the optimum aging process for the alloy is determined to be 200 ℃ for 16 h. Under an applied pressure of 42 MPa at 200 ℃, the time to peak aging is reduced from 16 h under conventional aging to 6 h, representing a 62.5% reduction, and the alloy exhibits a faster age-hardening response.

(2) Through extrusion of the AE45-Gd magnesium alloy, an as-extruded magnesium alloy containing both large blocky Al_2_RE (La, Ce, Gd) compounds and small granular Al_11_RE_3_ (La, Ce, Gd) particles was prepared. During the aging process, a pronounced Ostwald ripening phenomenon was observed, especially during high-temperature aging and the over-aging stage. The small granular Al_11_RE_3_ (La, Ce, Gd) particles appeared to gradually bridge and coalesce, and the large blocky Al_2_RE (La, Ce, Gd) compounds seemed to progressively engulf the small Al_11_RE_3_ (La, Ce, Gd) particles. In addition, aging under pressure appears to promote the Ostwald ripening process.

(3) After aging at 200 ℃ for 16 h, the AE45-Gd magnesium alloy achieves its optimal room-temperature mechanical properties, with a yield strength, tensile strength, and elongation reaching 186.36 (±4.5) MPa, 322.50 (±2.3) MPa and 4.32% (±0.52%), respectively. During the fracture process, microcracks predominantly initiate at the Al_2_RE (La, Ce, Gd) compounds. The Al_11_RE_3_ (La, Ce, Gd) particles show little evidence of self-cracking, and the cracking mainly occurs at the phase boundaries between these particles and the α-Mg matrix. With increasing load, the cracked Al_2_RE (La, Ce, Gd) and the cracked phase boundaries progressively interconnected, ultimately leading to the complete fracture of the alloy.

## Figures and Tables

**Table 1:** Chemical composition of AE45-Gd alloy (wt.%).

Alloy	Chemical Composition /wt.%					
	Al	La	Ce	Gd	Mn	Mg
AE45-Gd	4.04	3.65	1.56	1.77	0.05	bal.

**Table 2:** EDS point analysis results of AE45-Gd alloys.

EDS Position	Elements (at.%)					Possible Intermetallic Compound
	Mg	Al	La	Ce	Gd	
1	8.10	63.32	6.53	15.69	6.36	Al_2_RE (La, Ce, Gd)
2	78.69	16.25	3.25	1.71	0.07	Al_11_RE_3_ (La, Ce, Gd)
3	85.16	11.72	1.44	1.45	0.17	Al_11_RE_3_ (La, Ce, Gd)
4	10.49	59.57	6.83	15.73	7.38	Al_2_RE (La, Ce, Gd)
5	82.49	13.70	1.76	1.79	0.25	Al_11_RE_3_ (La, Ce, Gd)
6	88.51	8.95	1.56	0.90	0.04	Al_11_RE_3_ (La, Ce, Gd)

**Table 3:** Peak-aged hardness and time to peak hardness of AE45-Gd alloy.

Alloy	Peak Hardness (HV)				Time to Peak Hardness (h)			
	180 °C	200 °C	220	200 °C + 42 MPa	180 °C	200 °C	220	200 °C + 42 MPa
AE45-Gd	85.4	84.67	84.25	84.77	22	16	9	6

## Data Availability

The original contributions presented in this study are included in the article. Further inquiries can be directed to the corresponding author.
